# Application of Computational Simulation for Occupational Safety Assessment in a Psychiatric Hospital

**DOI:** 10.3390/ijerph22121811

**Published:** 2025-12-02

**Authors:** Ruan Lucas, Carmen Riascos, Eugenio Merino, Jader Borges

**Affiliations:** 1 Academic Unit of Mechanical Engineering, Federal University of Campina Grande, Campina Grande 58429-900, PB, Brazil; 2 Production Engineering Department, Federal University of Santa Catarina (UFSC), Florianópolis 88040-970, SC, Brazil; 3 Design Department, Graduate Program in Production Engineering and Graduate Program in Design, Federal University of Santa Catarina (UFSC), Florianópolis 88040-970, SC, Brazil

**Keywords:** nursing staff, occupational safety, workplace accidents, occupational risks, occupational simulation

## Abstract

Psychiatric hospitals present occupational hazards, with patients’ unexpected behaviors constituting a unique factor that makes the safety climate crucial for preventing injuries and ensuring a healthy work environment. In this context, this study analyzed occupational injuries and safety conditions in a psychiatric hospital in southern Brazil, employing system dynamics (SD)-based computational simulation as a decision-support tool. The study was carried out in five stages: definition of the case study; collection and descriptive analysis of injury data; development of safety indicators; SD modeling and simulation; and model validation through sensitivity analysis. The simulation results indicated that: (i) a 10% increase in personal protective equipment (PPE) usage reduced perceived risk to 76.48%; (ii) a 25% increase in patients’ unexpected behaviors resulted in six additional injuries; and (iii) standardizing 50% of activities could prevent seven injuries per year. The findings suggest that computational simulation enhances analytical capacity and demonstrate that the safety climate is directly influenced by mitigating occupational risks, implementing standardized internal procedures, training personnel, and establishing effective safety indicators.

## 1. Introduction

According to the International Labour Organization (ILO), workplace accidents represent a global concern due to their significant social and economic impacts [1]. In psychiatric hospitals, such accidents also occur, arising from workers’ exposure to physical, chemical, biological, ergonomic, and mechanical hazards (e.g., sharp objects) during routine professional activities [2,3,4].

In addition, professionals (e.g., nursing technicians, nurses) are exposed to patients’ aggressive behaviors, which constitute a unique occupational hazard rarely encountered in other work environments [5]. Consequently, violence-related incidents are a major concern for managers, particularly because they directly affect nurses and physicians [6,7].

Despite this concern, underreporting of aggression-related accidents is common in psychiatric hospitals [8]. This underreporting occurs for various reasons, such as professionals’ reluctance to report incidents or the perception that aggression is an inherent aspect of their work [4]. Whether reported or not, such incidents contribute to job dissatisfaction, which in turn may increase the risk of further violence [9,10].

In this context, the safety climate—defined as employees’ perceptions of organizational practices regarding occupational health and safety and overall risk perception—becomes a key factor [11,12,13]. A positive safety climate not only enhances job satisfaction but also reduces accidents [13,14]. Safety climate is directly influenced by managerial values and practices (e.g., adequacy of training, provision of protective equipment, quality of safety management systems), as well as internal communication and employee involvement in occupational health and safety [11,12,13].

Given this scenario, the research problem addressed in this study is how computational simulation can support decision-making to optimize the safety climate in psychiatric settings. Accordingly, the main objective is to analyze occupational injuries and safety conditions in a psychiatric hospital in southern Brazil and subsequently develop a computational simulation using system dynamics (SD).

This study is justified for three reasons: (i) a systematic literature review (SLR) revealed no studies applying computational simulation to analyze workplace injuries in psychiatric hospitals; (ii) SD is a computational method for quantitatively analyzing causal relationships over time, yet no scientific evidence was found of its application to injury analysis in psychiatric hospitals [4,15]; and (iii) the safety indicators developed in this study may assist other researchers in designing control mechanisms for safety management in similar work environments.

## 2. Materials and Methods

The development of this research was carried out in five stages. First, the case study site was selected. Next, data were collected and subjected to descriptive analysis. In the third stage, safety indicators were developed to enhance understanding of the existing conditions. Subsequently, System Dynamics (SD) was applied to (i) identify how variables were interrelated and influenced safety conditions, and (ii) structure the simulation model. Finally, the model was validated, followed by a sensitivity analysis to examine the effects of parameter variations on the variables.

### 2.1. Case Study Site

The study was conducted in a public hospital specializing in psychiatric care and chemical dependency, inaugurated on 10 November 1941. The hospital is located in southern Brazil, in the municipality of São José, state of Santa Catarina.

The research focused on the inpatient ward, and ethical approval was obtained from the Human Research Ethics Committee of the Federal University of Santa Catarina (No. 2595066). This ward was selected because it accounted for 71 accidents over the past seven years—representing 48% of all reported incidents—and was identified by hospital administration as the most critical unit in the facility.

A total of twenty-five nursing professionals, the occupational safety engineer, the safety technician, and the head nurse participated in this study. The details of their participation are provided in the following sections.

### 2.2. Data Collection and Descriptive Analysis

Ten technical visits were conducted to provide the researchers with a comprehensive understanding of the working conditions and dynamics in the inpatient ward. Simultaneously, additional relevant information for this study was collected.

To analyze the multiple causes of workplace injuries, a document review was performed using all Accident Reports (CAT) recorded by the occupational safety officer, as in previous studies [4]. Official records from the past seven years were analyzed, and three key pieces of information were extracted: injury date, occupation of the injured worker, and causal agent. The collected data were tabulated and stored using Microsoft Excel.

Further information regarding occupational injuries was obtained through targeted visits to the ward and structured interviews with the head nurse and the occupational safety engineer.

The information gathered during the interviews was categorized into four groups, based on previous studies [16,17,18]. The first group addressed organizational factors, specifically whether activities were supported by detailed Standard Operating Procedures (SOPs). The second group examined the relationship between personal protective equipment (PPE) use and injuries. The third group focused on organizational measures proposed for injury mitigation. Finally, the fourth group considered the level of PPE usage among staff.

To complement data collection, an exploratory questionnaire was designed and administered to capture the sociodemographic characteristics of the nursing staff, their perceptions of occupational risks, and self-reported workplace injuries. The questionnaire (Table A1) was based on previous research [19,20,21]. All 25 nursing staff members completed the questionnaire.

Injuries (Accident) incidence was examined from two sources: official records (CAT) and self-reports from the nursing staff. This dual approach aimed to identify possible underreporting, a common issue in psychiatric work environments [8,20].

Based on the collected data, initial descriptive analyses were performed. First, a general analysis of the hospital was conducted, identifying the total number of injuries, the most affected professionals, and the main causal agents. Subsequently, the analysis focused on the nursing staff, identifying the primary causes of injuries and staff perceptions of occupational risks.

At this stage, the safety technician contributed to the research by providing access to injury reports and clarifying any questions that arose. In addition, the safety perception of the twenty-five nursing professionals was assessed.

### 2.3. Occupational Safety Indicators

Based on on-site visits, document analysis, structured interviews, the questionnaire, and the initial descriptive analyses, occupational safety indicators were developed. The hospital previously lacked metrics for monitoring, which hindered effective evaluation of existing conditions, implemented measures, and their outcomes. Developing such indicators is particularly important given the recognized need for decision-support tools in occupational safety management [22].

The Multi-Criteria Decision Aid Constructivist (MCDA-C) methodology was employed, as it has been applied in both industrial and academic settings for the development of performance indicators [22,23]. This methodology emphasizes stakeholder involvement in the decision-making process and is structured in three phases: (1) problem structuring and indicator definition, (2) evaluation of potential actions, and (3) formulation of recommendations.

This study focused on problem structuring (Phase 1). It began with a meeting to identify the main stakeholders, which enabled the definition of Primary Evaluation Elements (PEEs)—in this case, the nursing staff working in the ward.

Subsequently, Action-Oriented Concepts (AOCs) were defined. These concepts guide desired actions and establish minimum expected outcomes for occupational safety aspects. They were derived through three approaches: (i) literature review; (ii) case study analysis; and (iii) perceptions of the stakeholders included in the PEEs. The concepts were grouped by area, represented in cognitive maps, and validated by the hospital’s occupational safety engineer.

Next, Strategic Objectives (SOs) were linked to each concept. For each SO, Elementary Points of View (EPVs) were defined, serving as the basis for constructing the indicators. Each indicator was assigned an ordinal scale to measure the level of compliance, and all indicators were validated by the hospital’s occupational safety engineer.

For easier interpretation, the indicators were classified into five categories according to their results: (i) very poor, 0–20% (red); (ii) poor, 21–40% (orange); (iii) intermediate, 41–60% (gray); (iv) good, 61–80% (light green); and (v) excellent, ≥81% (dark green).

Therefore, the development of the indicators, as well as their validation, involved the direct participation of the hospital’s occupational safety engineer. It is further emphasized that these indicators were subsequently used as parameters for constructing the simulation model.

### 2.4. Structuring of the Simulation Model

System Dynamics (SD) was employed to structure the simulation model. According to Lucas et al. [15], SD modeling comprises three main stages: causal definition, structural description, and quantification/projection.

(a) Causal definition: This stage describes the system using a Causal Loop Diagram (CLD), which illustrates relationships among variables through arrows indicating cause-and-effect links. A positive sign on the arrow denotes a direct relationship, while a negative sign indicates an inverse relationship [24].

In this study, the CLD was developed in Vensim software (version 10.1.5) based on information obtained from the preceding methodological steps. Constructing an effective CLD is essential for developing the Stock and Flow Diagram (SFD), which serves as the foundation for simulation modeling [25]. To ensure accuracy, the CLD was validated by both the hospital’s occupational safety engineer and the head nurse.

The results of this stage are presented concisely, as the main focus of this article is the simulation model. Therefore, the CLD outcomes are reported through the description of closed feedback loops, which is the standard approach for presenting this type of structure [15].

(b) Structural description: Mathematical modeling was used to develop the SFD, which includes all variables necessary to numerically describe the causal relationships identified in the previous stage, thereby enabling computational simulation [24].

The simulation was structured to capture the behavior of variables over time, based on stock and flow concepts. Stocks (also referred to as levels) represent conserved quantities in the system, while flows (also referred to as rates) are variables that modify those quantities [24,26].

As the modeling aimed to understand the system’s behavior over time, its mathematical foundation consisted of ordinary differential equations, assuming that stock levels result from inflows minus outflows [24,26].

The simulation model was designed to represent the system over one year, with monthly time steps, using the Euler integration method. It was developed in Vensim (version 10.1.5), and the variables, units, and mathematical equations are detailed in Table A2. Input variables for the model were defined based on descriptive analyses and the safety indicators.

(c) Quantification or projection: After consistency analysis and validation, the mathematical equations from the structural description were used to run computational simulations and evaluate variable behavior under parameter changes.

The model underwent a consistency analysis to assess the compatibility between the proposed structure and the system’s actual behavior. This evaluation was based on a ten-test checklist (Table A3), adapted from Lucas et al. [15]. In parallel, the model was validated by the hospital’s occupational safety engineer.

Once the model was calibrated, sensitivity analysis was performed to evaluate how parameter variations affected the variables.

### 2.5. Sensitivity Analysis

The purpose of the sensitivity analysis was to evaluate how the model responded to modifications in the parameters of specific variables. As shown in Table 1, the parameters of three variables were adjusted: (i) the rate of proper PPE usage; (ii) the rate of unexpected patient behaviors; and (iii) the rate of activities with established operational procedures.

The selection of parameters for adjustment was based on the scientific literature and the safety conditions identified in the case study. The aim of these simulations was to examine their impact on three key outcomes: overall perception of occupational risk, annual injury rate, and underreporting of injuries. All simulations were conducted using Vensim software.

It should be emphasized that only the variables presented in Table 1 were analyzed in the sensitivity analysis.

## 3. Results

The sample had a mean age of 36 years and an average length of service of 7 years. Of the 71 officially recorded injuries (accidents) involving nursing staff, 69% occurred with women and 31% with men. Furthermore, 46% (sample number = 33) of the injuries were due to aggression; 23% (sample number = 16) resulted from falls; 15% (n = 11) were caused by sharp objects; 10% (sample number = 7) were commuting injuries; and 6% (sample number = 4) were related to ergonomic factors.

Based on the administered questionnaires, it was found that 50% of the staff had previously experienced an injury, with self-reports totaling 93 incidents. The primary cause of self-reported injuries was aggression (sample number = 58; 62.37%), followed by falls (sample number = 23; 24.73%). Consequently, 22 self-reported injuries were not recorded in the official reports.

Regarding perceptions of occupational risks: (i) 100% of the respondents reported exposure to physical and psychological stress (ergonomic risk); (ii) 100% identified physical aggression as a constant risk (mechanical hazard); (iii) 93% reported the presence of bacteria in the environment (biological risk); (iv) 93% indicated exposure to tobacco smoke (cigarettes and straw) as a chemical risk; and (v) 71.4% reported exposure to noise.

### 3.1. Safety Indicators

The 23 proposed indicators provided a clearer understanding of the existing safety conditions. Overall, the results of six indicators (27%) were classified as “Good” or “Excellent,” five as “Intermediate” (23%), and 12 indicators (55%) were classified as “Poor” or “Very Poor”.

As shown in Table 2, “Storage quality”, “Quantity of stored products” and “Disease reports” were classified as “Excellent” (dark green). “Level of PPE protection provided,” “Availability of resources for equipment acquisition,” and “Level of PPE usage by staff” were classified as “Good” (light green).

At an intermediate level (gray), it was observed that half of the workers participating in safety training still used PPE incorrectly. This category also included the indicator assessing whether PPE use prevented contamination by bacteria, viruses, or protozoa.

Indicators classified as poor (orange) included “Number of staff using PPE correctly,” as well as: “Training provided for each PPE according to requirements,” “PPE with technical specification sheet to assess effectiveness,” and “Number of times workers checked PPE condition before use”.

Indicators classified as very poor (red) were: (i) “Rate of activities with described Standard Operating Procedures (SOPs),” (ii) “Rate of activities with written SOPs, including identified risks, PPE, and emergency procedures,” (iii) “Internal protocols to validate PPE,” and (iv) “Staff trained to verify PPE protection levels according to the risk of each activity”.

### 3.2. Identified Causal Relationships

**Figure 1:**
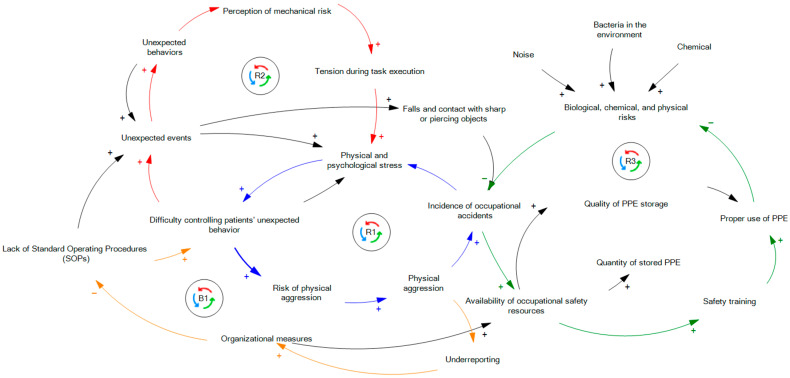
Causal loop diagram.

In the structured and validated Causal Loop Diagram (CLD), four loops were identified (Figure 1), which are represented by the different colors. The first loop (R1) showed that the absence of well-established standard operating procedures hindered the control of unexpected patient behaviors. This increased the risk of physical aggression and, consequently, the number of aggressive incidents in the ward. The occurrence of such injuries (accidents) elevated the stress levels of professionals, directly affecting the proper execution of procedures necessary for patient management.

The second loop (R2) highlighted that the incidence of unexpected patient behaviors directly influenced the nursing staff’s perception of high injury risk. This perception resulted in tension and a constant state of alertness during procedures, leading to peaks in stress. This mental state impaired professional performance, which, combined with the lack of well-established procedures, made patient management more difficult and increased the occurrence of unexpected behaviors.

The third loop (R3) demonstrated that greater availability of resources for the occupational safety area directly enhanced safety training. This, in turn, positively influenced the use of personal protective equipment (PPE) and mitigated the incidence of physical, chemical, and biological risks in the environment. The reduction in these hazards led to a lower incidence of injuries associated with them.

The fourth loop (B1) indicated that the incidence of physical aggression directly affected the underreporting rate. The higher the underreporting, the lower the hospital’s capacity to implement effective measures to mitigate the problem. Consequently, necessary procedures to manage patients in the face of unexpected behaviors, particularly to prevent aggression, were not implemented.

Finally, it was observed that incidents of physical aggression were directly related to the three main causes of injuries involving nursing staff: (i) aggression-related injuries; (ii) falls; and (iii) injuries involving sharp objects.

### 3.3. Computational Simulation

**Figure 2:**
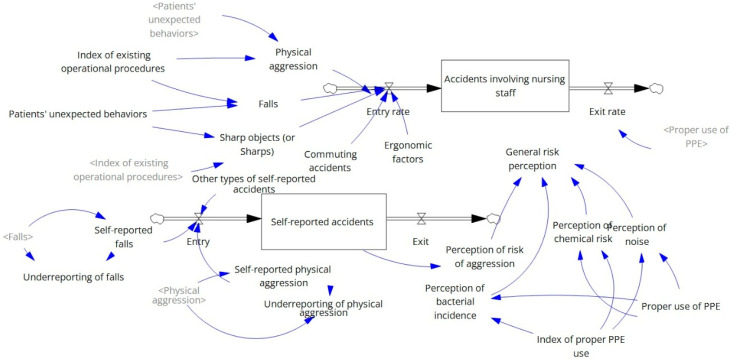
Stock and flow diagram (simulation model).

Figure 2 graphically represents the structured computational simulation model. Based on this model, the first simulation focused on the proper use of PPE, which was currently 40%, as identified in the safety indicator analysis. By increasing this rate by 10%, the nursing staff’s overall perception of occupational risk decreased from 89.35% to 76.48%, primarily due to changes in the perception of chemical, physical, and biological hazards. However, the perception of aggression risk remained unchanged, as did the injury (accident) rate among nursing staff.

**Figure 3:**
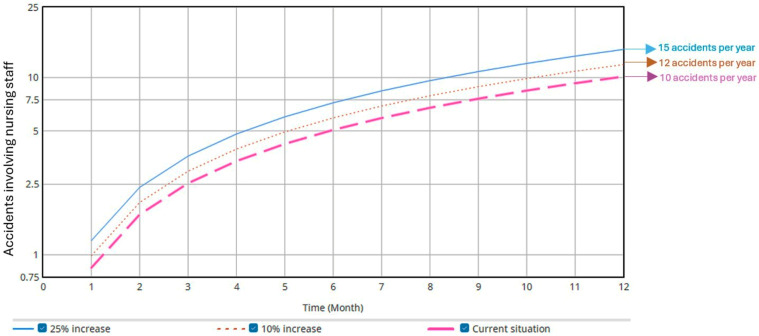
Influence of unexpected patient behavior on the accident rate.

**Figure 4:**
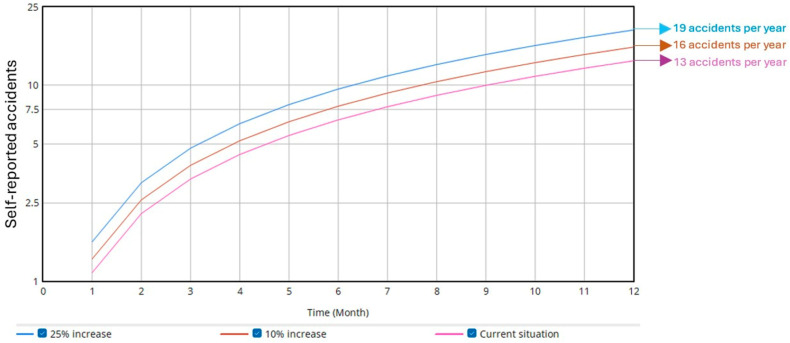
Influence of unexpected patient behavior on the self-reported accident rate.

The second simulation involved increasing the rate of unexpected patient behaviors by 10% and 25%. As shown in Figure 3, the annual injury (accident) rate increased by 1.68 (equivalent to two additional injuries (accidents) per year) and 4.28 (equivalent to five additional accidents per year), respectively. Additionally, due to underreporting, the rate of self-reported injuries (accidents) per year also increased (Figure 4) by 2.31 (equivalent to three additional accidents per year) and 5.78 (equivalent to six additional accidents per year), respectively.

**Figure 5:**
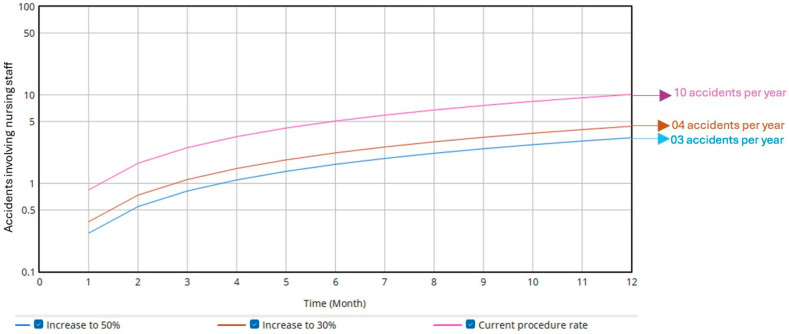
Influence of operational procedures on the accident rate.

The third simulation focused on the rate of existing operational procedures, which include the protocols required to manage unexpected patient behaviors. The simulation increased the current rate (10%) to 30% and 50%. As shown in Figure 5, the annual injury (accident rate decreased by 5.71 (equivalent to six fewer accidents per year) and 6.85 (equivalent to seven fewer accidents per year), respectively. Additionally, overall occupational risk perception decreased from 89.35% to 72.68% and 69.35%, respectively.

## 4. Discussion

When analyzing occupational injuries (accidents) involving nursing staff, physical aggression was identified as the primary cause. The occurrence of unexpected behaviors and acts of violence by patients has also been reported in previous studies [4,19,27], highlighting the need to prioritize this issue to ensure occupational safety in these environments.

However, since aggression is recurrent, many affected professionals prefer not to report such incidents, either out of fear of punishment or being held responsible [8,20]. This was confirmed in the present analysis, as 22 injuries were self-reported by staff but not recorded in the hospital’s official records.

Regarding safety-related aspects, positive indicators were observed for personal protective equipment (PPE), particularly in terms of quality, storage, and distribution. Nevertheless, there was a clear need to increase proper PPE use and, importantly, to expand operational procedures for performing activities.

Focusing safety efforts on the acquisition and proper use of PPE is crucial to prevent various types of hazards, including physical, chemical, and biological risks. However, this alone is insufficient, as the main sources of accidents in this type of environment stem from patient behavior and aggression [5,6,16].

It was observed that, although the hospital emphasized preventive measures, it lacked strategies that could significantly impact the safety climate, enabling professionals to feel more confident in managing patient behaviors and, importantly, in reporting all incidents of aggression and violence. This is particularly relevant, as measures that directly influence the safety climate have been shown to be more effective in the long term [12].

The increase in unexpected patient behaviors directly affected both the injury (accident) rate and underreporting, as identified through computational simulation. For example, the results showed that a 25% increase in unexpected behaviors could result in up to six additional injuries (accidents) per year, highlighting the risks posed to staff in the absence of new control measures [4].

In this context, the existence of well-established protocols for managing these behaviors is essential [28,29]. Expanding documentation of activities through the implementation of Standard Operating Procedures (SOPs) is therefore critical. It was identified that only 10% of activities currently had such procedures. If this indicator were increased to 50% of activities, for instance, the annual number of injuries (accidents) could be reduced by seven, and overall risk perception would be directly improved.

Thus, the introduction of SOPs, followed by well-defined training protocols, demonstrates organizational support for daily practices, enhances staff’s sense of safety, and is essential for controlling and mitigating incidents of violence and aggression in psychiatric settings [6,17].

The simulation results, therefore, enhanced analytical capacity regarding safety conditions and reinforced the need for critical actions to ensure health and well-being. These include clearly defined procedures for control and containment, as well as specialized training in conflict resolution strategies, which should be incorporated into the SOP for each activity. These findings align with previous studies [15,20], which highlighted (i) the importance of computational simulation for optimizing workplace conditions, and (ii) how well-defined protocols can assist nurses in managing unexpected behaviors and physical aggression.

## 5. Conclusions

The nursing staff at the psychiatric hospital was exposed to occupational risks, with physical aggression identified as the primary cause of injuries and a constant mechanical hazard. Although the hospital demonstrated concern regarding the use of personal protective equipment (PPE), effective measures to increase compliance remain necessary. Moreover, the formalization of procedural protocols was identified as a key need, aiming to standardize internal actions that enhance safety during task execution.

The computational simulation indicated that increasing proper PPE use contributed to a reduction in the nursing staff’s overall perception of occupational risk, particularly regarding chemical, physical, and biological hazards. Conversely, an increase in unexpected patient behaviors directly led to a higher incidence of injuries, highlighting this factor as a significant aggravator of working conditions. Increasing the rate of operational procedures, including protocols for managing such behaviors, proved to be the most effective intervention, as it reduced both the number of injuries and perceived risk.

The results obtained from the computational simulation enhanced analytical capacity and demonstrated that the safety climate in the psychiatric hospital is directly influenced by four main factors: (i) work environments that minimize exposure to hazards; (ii) well-established internal procedures for managing diverse situations, particularly those addressing actions in response to unexpected patient behaviors; (iii) a team trained according to internal procedures and aware of the importance of preventive actions; and (iv) effective internal indicators regarding the storage, validity, training, and use of PPE.

The main limitation of this study was the lack of temporal availability to implement the actions proposed in the simulation, particularly regarding the integration of operational procedures into daily practice. In addition, the nursing team sample consisted of 25 staff members. Therefore, future research is suggested to: (i) develop studies that simulate safety conditions in psychiatric hospitals, subsequently implement the proposed actions, and perform practical verification; and (ii) use computational simulation to better understand the dynamics of aggression-related injuries, aiming to identify all relevant variables and causal relationships, as well as factors that could directly contribute to mitigating these incidents.

## Figures and Tables

**Table 1:** Simulations performed.

Variable	Simulation Performed	Rationale
Proper PPE Utilization Rate	Increase of 10% in the current indicator, from 40% to 50%	Increasing this level of utilization is one of the managers’ primary objectives; therefore, it is important to understand the impacts of this increase.
Unexpected Patient Behavior	Increase in the unexpected patient behavior rate by 10% and 25%	The number of patients may increase, and random behaviors cannot be predicted; therefore, an increase in such behaviors is inherent to the work environment
Rate of Activities with Established Standard Operating Procedures (SOPs)	Increase in the current rate of activities with established SOPs (10%) to 30% and 50%	The absence of established procedures compromises professionals’ performance in situations involving unexpected behaviors and physical aggression from patients; therefore, understanding the impacts of this increase on occupational safety becomes essential.

**Table 2:** Proposed safety indicators and results obtained.

Indicator	Description	Result (%)
1. Storage	Quality of PPE storage (temperature, humidity, ventilation, weight)	100
2. Stock	Available stock for each PPE	100
3 Disease reports	% of investigated diseases where the use of PPE influences contamination	100
4. PPE provided	PPE provided that properly protects against occupational risks	80
5. Training 1	Training planned for each PPE vs. required training	75
6. Availability	Availability of resources to purchase PPE	75
7. PPE usage	Staff using PPE during occupational activities	70
8. PPE-Related illnesses	Investigated diseases where PPE use did not affect contamination	70
9. Training 3	Training included in schedule vs. Training required for each PPE	60
10. PPE assessment	Timing of PPE assessment according to occupational activity	60
11. Lack of PPE usage training	Staff required to attend training but unable due to lack of replacement	60
12. PPE usage training	Workers who attended training but use PPE incorrectly	50
13. Accidents related to PPE use	Accidents where PPE use did not affect contamination	45
14. Comfort level	PPE comfort level assessed in the last semester	40
15. Proper PPE usage	Staff properly using PPE during occupational activities	40
16. Training 2	Training provided for each PPE according to requirements	35
17 Technical specification sheet	PPE with technical specification sheet to assess effectiveness	30
18. PPE condition check	Number of times workers Check PPE condition before use	30
19. Protocols	Protocols defined to validate PPE	20
20. Updated SOP	SOP under update, including occupational risks, prevention activities, and PPE in writing	10
21. Described SOP	Occupational activities with written SOPs, including identified risks and PPE	0
22. Activities with SOP	Occupational activities with described SOP	0
23. Inspection	Number of staff trained to verify PPE protection levels according to activity risk	0

## Data Availability

The data supporting the conclusions of this article can be made available by the authors upon request.

## Appendix A

**Table A1:** Exploratory questionnaire. Reprinted from ref. [4].

Question
What is your email address?
Do you agree with the Free and Informed Consent Form (FICF)?
What is your gender?
How old are you?
What is your weight?
What is your height?
Are you a practitioner of physical activities?
Are you a smoker?
Do you have a chronic or degenerative disease? Which?
What is your marital status?
Do you have children?
What is your work schedule?
What is your education level?
What is your assignment?
What is your professional time?
Working time at a psychiatric hospital?
Describe your activities throughout the working day.
Do you take breaks during your workday?
Is there an appropriate place to rest?
Indicate the physical hazards you believe you are exposed to.
Indicate the chemical hazards you believe you are exposed to.
Indicate the biological hazards you believe you are exposed to.
Indicate the ergonomic hazards you believe you are exposed to.
Indicate the risks of accidents you believe you are exposed to.

**Table A2:** Simulation equations.

Variable	Acronym	Equation	Unit
Accidents involving nursing staff	AINS	$\mathrm{AINS}=\int_{to}^{t}[CA+PA+EF+SO+SO]+{AINS}_{to}$	Accidents/month
Self-reported accidents	SRA	$\mathrm{SRA}=\int_{to}^{t}[SPA+OTSA+SRF]+{SRA}_{to}$	Self-reported/month
Physical aggression	PA	(PUB/IEOP)/CONST	PA/month
Falls	FA	(PUB/IEOP)/CONS	FA/month
Sharp objects (or Sharps)	SO	(PUB/IEOP)/COS	SO/month
Self-reported falls	SRF	FA/0.695652	SRF/month
Underreporting of falls	UF	1-(FA/SRF)	UF/month
Self-reported physical aggression	SPA	PA/0.568965	SPA/month
Underreporting of physical aggression	UPA	1-(PA/SPA)	UPA/month
Perception of risk of aggression	PRA	(PUB/IEOP)/5	Rate
Perception of chemical risk	PCR	0.372/PPPE	Rate
Perception of noise	PN	0.2856/PPPE	Rate
Perception of bacterial incidence	PBI	0.372/PPP	Rate
General risk perception	GRP	(PRA + PN + PCR + PBI)/4	Rate
Index of existing operational procedures	IEOP	Constant	dmnl
Patients’ unexpected behaviors	PUB	Constant	dmnl
Indicator of physical aggression	CONST	Constant	dmnl
Falls indicator	CONS	Constant	dmnl
Indicator regarding sharps accidents	COS	Constant	dmnl
Commuting accidents	CA	Constant	CA/month
Ergonomic factors	EF	Constant	EF/month
Other types of self-reported accidents	OTSA	Constant	OTSA/month
Proper use of PPE	PPPE	Constant	dmnl

**Table A3:** Model Validation Checklist. Reprinted from ref. [15].

Model Validation Checklist
1. Structure Assessment
☐ Verify that the model structure is consistent with relevant descriptive knowledge of the system.
☐ Check whether the level of aggregation is appropriate.
☐ Assess whether decision rules adequately capture the behavior of actors in the system.
☐ Conduct partial model tests to evaluate the intended rationality of decision rules.
☐ Develop disaggregated submodels and compare their results with aggregated formulations.
☐ Disaggregate suspicious structures and repeat sensitivity and policy analyses.
2. Dimensional Consistency
☐ Test all equations using dimensional analysis software.
☐ Inspect equations for parameters that lack real-world meaning.
3. Parameter Assessment
☐ Confirm that parameter values are consistent with descriptive and numerical knowledge of the system.
☐ Verify that all parameters have real-world counterparts.
☐ Use partial model tests to calibrate subsystems.
☐ Apply judgment-based methods (interviews, expert opinions, focus groups, archival materials, direct experience).
☐ Develop disaggregated submodels to estimate relationships supporting aggregated models.
4. Extreme Conditions
☐ Inspect each equation to ensure plausibility under extreme inputs.
☐ Test the model’s response to extreme values of each variable, both individually and in combination.
☐ Subject the model to large shocks and extreme conditions.
5. Integration Error
☐ Halve the time step and observe behavioral changes.
☐ Use alternative numerical integration methods and compare results.
6. Behavior Reproduction
☐ Verify whether the model reproduces system behavior qualitatively and quantitatively.
☐ Assess whether the model generates the observed modes of system behavior.
☐ Compare frequencies and phase relationships between simulated variables and real-world data.
☐ Compute statistical measures of fit between model output and empirical data.
☐ Compare qualitatively the shapes of variables, asymmetries, amplitudes, unusual events, and relative phases.
☐ Examine model responses to test inputs, shocks, and noise.
7. Behavioral Anomalies
☐ Perform loop knockout analysis (zeroing major effects).
☐ Replace equilibrium assumptions with disequilibrium structures and examine responses.
8. Replication
☐ Test whether the model replicates behavior across different instances of the same system.
☐ Calibrate the model for the widest possible variety of related systems.
9. Surprise Behavior
☐ Maintain accurate, complete, and dated records of simulations.
☐ Verify whether the model generates previously unrecognized behaviors.
☐ Use the model to simulate system responses under new conditions.
☐ Investigate and resolve discrepancies between model behavior and system understanding.
10. Sensitivity Analysis
☐ Perform univariate sensitivity analyses (varying one parameter at a time).
☐ Perform multivariate sensitivity analyses (varying multiple parameters simultaneously).
☐ Test behavioral sensitivity: assess whether generated modes of behavior change significantly.
☐ Test policy sensitivity: evaluate whether policy implications change significantly when assumptions about parameters, limits, and aggregation vary within plausible uncertainty ranges.
☐ Use optimization methods to identify the best parameters and policies.
☐ Use optimization methods to identify parameter combinations that generate implausible results or policy reversals.
